# Central Hypoventilation Is a Key Risk Factor for Mechanical Ventilation During the Acute Phase of Anti-N-Methyl-D-Aspartate Receptor Encephalitis

**DOI:** 10.3389/fneur.2021.728594

**Published:** 2021-11-02

**Authors:** Qianhui Xu, Qian Wang, Jing Han, Fengju Mao, Silin Zeng, Siyan Chen, Chenyong Zhao, Mei Gu, Zaiwang Li, Xuejun Fu, Xiaoguang Luo, Ying Huang

**Affiliations:** Department of Neurology, Shenzhen People's Hospital (The Second Clinical Medical College, Jinan University, The First Affiliated Hospital, Southern University of Science and Technology), Shenzhen, China

**Keywords:** anti-N-methyl-D-aspartate receptor encephalitis, autoimmune encephalitis, central hypoventilation, mechanical ventilation, risk factors

## Abstract

**Objective:** Anti-N-methyl-D-aspartate receptor (anti-NMDAR) encephalitis is an acute form of encephalitis of autoimmune etiology. We aimed to evaluate the risk factors that predicted the need for mechanical ventilation during the acute phase of anti-NMDAR encephalitis through an analysis of the clinical characteristics and biochemical test results of the patients with anti-NMDAR encephalitis.

**Methods:** In this retrospective study, patients who primarily presented with anti-NMDAR encephalitis and exhibited anti-NMDAR antibody positivity in the cerebrospinal fluid (CSF) between November 2015 and February 2020 were included. Data on the clinical characteristics, biochemical test results, and treatment methods selected for the patients were collected for the analysis of factors predicting the need for mechanical ventilation.

**Results:** Thirty-one patients with a median age of onset of 31 years (inter-quartile range: 21–48 years) were included in this study, of which 15 were male (48.4%). Psychosis (23, 74.2%), seizures (20, 64.5%), and memory deficit (20, 64.5%) were the most common clinical manifestations. At admission, 17 patients (54.8%) presented with pyrexia, of which 12 (38.7%) had a body temperature ≥38°C, and six patients (19.4%) presented with central hypoventilation. All patients received first-line therapy (glucocorticoids, intravenous immunoglobulin, or plasmapheresis alone or combined), whereas two patients (6.5%) received rituximab, a second-line agent, as well. Seven patents required mechanical ventilation. Results of univariate logistic regression analysis revealed that body temperature ≥38°C [odds ratio (OR) = 18, 95% confidence interval (CI): 1.79–181.31, *P* < 0.05] and central hypoventilation at admission (OR = 57.50, 95% CI: 4.32–764.89, *P* < 0.05) were the risk factors for mechanical ventilation. Multivariate logistic regression analysis showed that central hypoventilation at admission was the only risk factor predicting the need for mechanical ventilation.

**Conclusion:** Central hypoventilation at admission is a key risk factor for mechanical ventilation during hospitalization in patients with anti-NMDAR encephalitis.

## Introduction

Anti-N-methyl-D-aspartate receptor (anti-NMDAR) encephalitis is the most common form of autoimmune encephalitis and is associated with autoantibodies against neuronal surface or synaptic antigens (1, 2). Since the first report of anti-NMDAR encephalitis in 2007 (3), the number of confirmed diagnoses has increased rapidly with the widespread application of detection methods. Differences in the clinical characteristics and treatment strategies for anti-NMDAR encephalitis among patients of different races and countries have also been reported (4). A study conducted on patients of Peking Union Medical College Hospital revealed that 20% of patients with anti-NMDAR encephalitis required mechanical ventilation (5). The reported mortality rates of anti-NMDAR encephalitis range from 2.7 to 11.45% (6). Among the various factors, early treatment, and the patient not requiring admission to the intensive care unit (ICU) are the predictors of good prognosis (7). In the present study, we retrospectively reviewed the data of patients, who had been referred to the Neurology Center of Shenzhen People's Hospital and diagnosed with anti-NMDAR encephalitis, for the investigation and analysis of predictors of mechanical ventilation during the acute phase of anti-NMDAR encephalitis.

## Materials and Methods

We retrospectively reviewed the medical records of the Neurology Center of Shenzhen People's Hospital from November 2015 to February 2020. All subjects included were adults. The inclusion criteria were as follows: ➀ Acute onset of one or more of the following types of manifestations: psychosis, memory deficit, speech disturbance, seizures, movement disorder, loss of consciousness, autonomic dysfunction, and central hypoventilation; ➁ Anti-NMDAR antibody positivity in the cerebrospinal fluid (CSF) by a cell-based assay (EUROIMMUN, Lübeck, Germany); ➂ Reasonable exclusion of other disorders (1). The following data of the included patients were recorded in detail: demographic (including sex and age) and clinical characteristics, CSF analysis results, brain magnetic resonance imaging (MRI) and electroencephalography (EEG) findings, absence or presence of tumors, time between onset and treatment, and the need for mechanical ventilation. Sleep disorders include insomnia, hypersomnia, and sleep pattern inversion, excluding behavioral sleep disorders. Central hypoventilation on admission is defined as alveolar hypoventilation that occurs while awake and meets one of the following conditions: (1) The arterial partial pressure of carbon dioxide (PaCO_2_) in the arterial blood gas analysis >45 mmHg. (2) The saturation of peripheral oxygen (SpO_2_) in room air/no supplemental O_2_ <95% (8), excluding cases of COPD, asthma, lung infection, status epilepticus, and drug sedation that can cause hypercapnic hypoventilation. The subjects were divided into two groups according to their need for mechanical ventilation, namely the critical illness group consisting of patients who required mechanical ventilation and the mild illness group consisting of patients who did not require mechanical ventilation. Risk factors for acute critical illness in which mechanical ventilation was required were analyzed and evaluated. The study was approved by the Research Ethics Committee of Shenzhen People's Hospital (ID: LL-KY-2021646). Written informed consent was obtained from each subject.

### Statistical Analysis

Symptom frequencies were expressed as percentages. Demographic characteristics and the number of days from onset to commencement of first-line therapy were expressed as median and interquartile range (IQR). Clinical variables of the patients with critical illness (requiring mechanical ventilation) and mild illness (not requiring mechanical ventilation) were compared using univariate logistic regression analysis, and variables with *P* < 0.05 were included in the subsequent multivariate logistic regression analysis. Differences were considered statistically significant when *P* < 0.05.

## Results

### Clinical Characteristics and Treatment of Patients

We retrospectively analyzed the data of 31 patients diagnosed with anti-NMDAR encephalitis. Table 1 shows the clinical characteristics of these patients. The median age was 31 years (IQR: 21–48 years) and women slightly outnumbered men (16 women, 51.6%). Nine patients (29%) had a history of prior infection. The main clinical symptoms were psychosis (23 patients, 74.2%), seizures (20 patients, 64.5%), memory deficit (20 patients, 64.5%), decreased level of consciousness (13 patients, 41.9%), movement disorder (8 patients, 25.8%), sleep disorder (8 patients, 25.8%), and speech disturbance (7 patients, 22.6%). Eleven patients experienced headache (35.5%) and the incidence rate of headache in patients with central hypoventilation was 4/6 (66.7%), which was significantly higher than that in patients without central hypoventilation (7/25, 28%). Eleven patients (35.5%) had abnormal brain MRI findings and 16 patients (51.6%) had abnormal EEG findings, with slow activity being the most common EEG abnormality (14 patients, 45.2%). Twenty-one patients had an increased CSF cell count (67.7%) and eight patients had an increased CSF protein level (25.8%). Anti-NMDAR antibody testing had been performed on the serum and CSF samples of all patients, and anti-NMDAR antibody positivity was detected in all CSF samples. All female patients underwent gynecologic ultrasonography or pelvic computed tomography scanning, which revealed the presence of ovarian teratoma in four patients (4/16, 25%). All patients received first-line therapy (glucocorticoids, intravenous immunoglobulin, or plasmapheresis alone or combined), while two patients (6.5%) received rituximab, a second-line agent, as well.

**Table 1:** Clinical characteristics of patients with anti-NMDAR encephalitis.

**Clinical characteristics**	**All (*n* = 31)**
Median age (IQR)	31 (21-48)
Sex (male) (*n*, %)	15 (48.4)
Symptoms	
Prodromal symptoms (*n*, %)	9 (29)
Psychosis (*n*, %)	23 (74.2)
Positive symptoms of psychosis (*n*, %)	19 (61.3)
Negative symptoms of psychosis (*n*, %)	13 (41.9)
Seizures (*n*, %)	20 (64.5)
Pyrexia ≥37.3°C (*n*, %)	17 (54.8)
Body temperature ≥38°C (*n*, %)	12 (38.7)
Decreased level of consciousness (*n*, %)	13 (41.9)
Memory deficit (*n*, %)	20 (64.5)
Speech disturbance (*n*, %)	7 (22.6)
Movement disorder (*n*, %)	8 (25.8)
Sleep disorder (*n*, %)	8 (25.8)
Central hypoventilation at admission (*n*, %)	6 (19.4)
Limb weakness (*n*, %)	2 (6.5)
Headache (*n*, %)	11 (35.5)
Admission to the ICU (*n*, %)	7 (22.6)
Mechanical ventilation (*n*, %)	7 (22.6)
Arrhythmia (*n*, %)	8 (25.8)
Ovarian teratoma (*n*, %)	4 (25%)
Treatment and prognosis	
First-line therapy (*n*, %)	31 (100)
Days from onset to treatment, median (IQR)	13 (2-21)
Early immunotherapy (≤15 days) (*n*, %)	13 (41.9)
Second-line therapy (*n*, %)	2 (6.5)
Relapsed patients (*n*, %)	4 (12.9)
Abnormal MRI findings (*n*, %)	11 (35.5)
EEG findings	
Abnormal EEG findings (*n*, %)	16 (51.6)
Epileptic discharges (*n*, %)	7 (22.6)
Slow activity (*n*, %)	14 (45.2)
CSF findings	
CSF protein ≥0.45 mg/dl (*n*, %)	8 (25.8)
CSF cell count >5 cells/μl (*n*, %)	21 (67.7)
Baseline mRS score >2 (*n*, %)	26 (83.9)

*Anti-NMDAR, anti-N-methyl-D-aspartate receptor; IQR, interquartile range; ICU, intensive care unit; MRI, magnetic resonance imaging; EEG, electroencephalogram; CSF, cerebrospinal fluid; mRS, modified Rankin score*.

### Outcome and Predictive Factors

Seven patients in our cohort ultimately required mechanical ventilation during their hospital stay. Table 2 shows the results of univariate logistic regression analysis for patients who required mechanical ventilation. It was found that body temperature ≥38°C [odds ratio (OR) = 18, 95% confidence interval (CI): 1.79–181.31, *P* < 0.05] and central hypoventilation at admission (OR = 57.50, 95% CI: 4.32–764.89, *P* < 0.05) were the risk factors for mechanical ventilation. Differences in other factors such as age at onset, time from onset to immunotherapy, sex, tumor association, CSF status, MRI findings, and EEG findings were not statistically significant. Multivariate logistic regression analysis showed that central hypoventilation at admission (OR = 57.50, 95% CI: 4.32–764.89, *P* < 0.05) was the only risk factor for mechanical ventilation.

**Table 2:** Univariate logistic regression analysis of risk factors predicting the need for mechanical ventilation.

**Clinical characteristics**	***P*** **-value**	**Odds ratio (95% CI)**
Age (years)	0.22	0.96 (0.89–1.03)
Male	0.07	8.40 (0.87–81.08)
Symptoms		
Seizures	0.21	4.29 (0.44–41.37)
Body temperature ≥38°C	0.01	18.0 (1.79–181.31)
Psychosis	0.44	2.47 (0.25–24.46)
Decreased level of consciousness	0.12	0.17 (0.02–1.60)
Memory deficit	0.21	4.29 (0.44–41.37)
Speech disturbance	0.67	1.52 (0.22–10.30)
Movement disorder	0.85	1.20 (0.18–7.88)
Sleep disorder	0.25	2.85 (0.48–17.10)
Headache	0.19	3.24 (0.57–18.39)
Central hypoventilation	0.00	57.5 (4.32–764.89)
Ovarian teratoma	0.18	4.40 (0.49–39.21)
CSF analysis of acute phase		
Protein >45 mg/dl	0.85	1.20 (0.18–7.88)
WBC >5 cells/μl (*n*, %)	0.27	3.6 (0.37–34.94)
Abnormal EEG findings	0.74	1.33 (0.24–7.28)
Abnormal brain MRI signal	0.21	0.23 (0.02–2.25)
Time to treatment	0.68	0.99 (0.92–1.06)
Early immunotherapy (<15 days)	0.96	1.05 (0.19–5.76)

*IQR, interquartile range; CI, confidence interval; MRI, magnetic resonance imaging; EEG, electroencephalogram; CSF, cerebrospinal fluid; WBC, white blood cells*.

## Discussion

In the present study, we retrospectively analyzed 31 cases of anti-NMDAR encephalitis that had characteristic clinical features and were diagnosed through CSF antibody testing. Symptoms generally exhibited by the patients included psychosis, cognitive disorder, seizures, and movement disorders. Eleven patients (35.5%) had abnormal MRI findings. This is in agreement with the results of a systematic review, which reported that fewer than half of the patients with anti-NMDAR encephalitis had abnormal findings on MRI (9). Electroencephalogram abnormalities are extremely common in anti-NMDAR encephalitis. A recent large sample study of Chinese patients with anti-NMDAR encephalitis by Xu et al. found that 51.4% of the patients had abnormal EEG findings (5), consisting mainly of non-specific slow activity changes. This is consistent with our results, which indicated the presence of EEG abnormalities in 51.6% of the subjects.

Although most patients with anti-NMDAR encephalitis have a good prognosis, a certain proportion of patients eventually become critically ill. There are a number of proven risk factors for poor long-term outcome including ICU admission (7), low Glasgow Coma Score at admission (2, 10), and abnormal EEG at admission (11). Complications during hospitalization, such as pneumonia and respiratory failure, affect the outcomes and increase patient mortality (10, 12). The use of mechanical ventilation also leads to other complications such as hospital-acquired pneumonia. In a retrospective study, it was also found that patients with anti-NMDAR encephalitis accounted for 1% of young patients admitted to the ICU (13). In the aforementioned study by Xu et al., it was reported that approximately 20% of the patients with anti-NMDAR encephalitis required mechanical ventilation (5), which is consistent with our results (22.6%). The median duration of ventilatory support in central insufficiency by Dalmau et al. was 8 weeks, with a range of 2–40 weeks (14). The need for mechanical ventilation in anti-NMDAR encephalitis is indicative of disease severity, and the results of univariate logistic analysis in the present study showed that pyrexia ≥38°C and central hypoventilation were risk factors for of mechanical ventilation.

Acquired central hypoventilation is associated with brainstem tumors, ischemia, and Chiari malformation. Previous research has reported the presence of central hypoventilation in anti-Hu-associated paraneoplastic encephalomyelitis (15) and anti-NMDAR encephalitis (16). Destruction of brainstem respiratory nuclei, which include the dorsal respiratory group within the nucleus tractus solitarius, pontine respiratory group, and ventral respiratory group, is thought to be the possible mechanism of central hypoventilation (17). The type of nuclear groups affected may selectively affect either automatic or voluntary respiration and determine the treatment method (17). Autopsy results of a patient with anti-Hu-associated central hypoventilation revealed the presence of pathological changes in these brainstem regions, widespread perivenous and parenchymal inflammatory infiltrates, and severe neuronal loss in the medulla involving the nuclei of the 12^th^ cranial nerve, inferior olives, nucleus ambiguous, and reticular system (18).

The incidence of hypoventilation in the course of NMDAR encephalitis reported by Dalmau et al. is very high (66/100) (14). The proportion of hypoventilation in our report is much lower. The possible reasons are as follows: First, we only counted hypoventilation during admission; second, some patients with central hypoventilation have normal ventilation while awake but hypoventilate when asleep. However, patients with central hypoventilation while awake were included in our study; and third, the proportion of teratomas in our report was low (25 vs. 53%). Anti-NMDAR-associated hypoventilation may share a common pathogenesis with that associated with other forms of encephalitis. Teratoma-associated encephalitis is an extensive or multifocal encephalitis in which brainstem involvement is frequent, which may be the cause of hypoventilation (16). The ubiquitous distribution of the NR1 subunit of the NMDA receptor in the brain (19–21), may be a possible cause of the higher tendency of central hypoventilation in anti-NMDAR encephalitis compared with that in other forms of encephalitis. In addition, up to 3/4 of patients have prodromal symptoms, including fever, headache, nausea, vomiting, and upper respiratory tract infection (22). These initial infection events can make the blood-brain barrier more prone to damage (23, 24). They trigger and enhance the immune process (25), which may be the reason for the higher need for mechanical ventilation in patients with pyrexia.

Central hypoventilation can be managed by non-invasive ventilation, but this is highly dependent on patient compliance. Treatment methods for congenital central hypoventilation syndrome include non-invasive ventilation, diaphragm pacing by phrenic nerve stimulation, tracheotomy, and domiciliary ventilation. Management of central hypoventilation in brainstem encephalitis using phrenic nerve pacing has also been reported (26). The treatment of hypoventilation related to NMDAR encephalitis can learn from the treatment of congenital hypoventilation. As anti-NMDAR antibodies bind to the extracellular domains of cell surface proteins, the neuronal dysfunction is usually reversible (27). Therefore, early immunotherapy is beneficial for the treatment of anti-NMDAR encephalitis (28). In the present study, early immunotherapy did not lead to a significant decrease in the need for mechanical ventilation. This may be attributed to the fact that the median duration of treatment was 13 days, which means that most patients had received early intervention. Another reason may be related to our low overall number of cases. Among the four patients with ovarian teratomas, two required mechanical ventilation which was not attributable to gynecologic surgery. Although the difference was not statistically significant, this result seems to suggest that patients with teratomas may have a higher likelihood of requiring mechanical ventilation. Therefore, we recommend surgical excision of teratomas at the earliest possible opportunity.

In conclusion, central hypoventilation at admission is a key predictor of the need for mechanical ventilation in patients with anti-NMDAR encephalitis. This suggests that early identification of central hypoventilation is required for the prompt elimination of possible causes that could lead to its aggravation. Our study has several limitations. As this was a retrospective single-center study, evaluation of clinical data was solely based on the data recorded by the clinicians. In our study, the assessment of central hypopnea was approximate. Therefore, prospective studies based on standard blood gas analysis and polysomnography are needed. Due to the small sample size of this study, further studies with large samples are required for the validation of our results.

## Data Availability Statement

The raw data supporting the conclusions of this article will be made available by the authors, without undue reservation.

## Ethics Statement

The studies involving human participants were reviewed and approved by Ethics Committee of Shenzhen People's Hospital. The patients/participants provided their written informed consent to participate in this study.

## Author Contributions

YH and QX concepted, designed, and supervised the study. QW, JH, FM, SZ, SC, CZ, and MG acquired the data. QX analyzed and interpreted the data, provided statistical analysis, had full access to all of the data in the study, and are responsible for the integrity of the data and the accuracy of the data analysis and drafted the manuscript. ZL, XF, and XL critically revised the manuscript for important intellectual content. All authors contributed to the article and approved the submitted version.

## Conflict of Interest

The authors declare that the research was conducted in the absence of any commercial or financial relationships that could be construed as a potential conflict of interest.

## Publisher's Note

All claims expressed in this article are solely those of the authors and do not necessarily represent those of their affiliated organizations, or those of the publisher, the editors and the reviewers. Any product that may be evaluated in this article, or claim that may be made by its manufacturer, is not guaranteed or endorsed by the publisher.

## Abbreviations

anti-NMDAR: anti-N-methyl-D-aspartate receptor
CSF: cerebrospinal fluid
MRI: magnetic resonance imaging
EEG: electroencephalogram
OR: odds ratio
CI: confidence interval
IQR: interquartile range.
